# MISMARPE protocol: minimally invasive surgical and miniscrew-assisted rapid palatal expansion

**DOI:** 10.1590/2177-6709.29.3.e24spe3

**Published:** 2024-08-09

**Authors:** Orion Luiz HAAS, Paulo Ricardo Baccarin MATJE, Bibiana Mello da ROSA, Vicente Dias PICCOLI, Susana Maria Deon RIZZATTO, Rogério Belle de OLIVEIRA, Luciane Macedo de MENEZES

**Affiliations:** 1 Pontifical Catholic University of Rio Grande do Sul, School of Health and Life Sciences, Postgraduate Program in Dentistry (Porto Alegre/RS, Brazil).; 2 Pontifical Catholic University of Rio Grande do Sul, School of Health and Life Sciences, Department of Orthodontics (Porto Alegre/RS, Brazil).; 3 São Lucas Hospital of Pontifical Catholic University of Rio Grande do Sul, Department of Oral and Maxillofacial Surgery (Porto Alegre/RS, Brazil).; 4 Private Practice (Porto Alegre/RS, Brazil).

**Keywords:** Orthodontics, Minimally invasive surgical procedures, Orthognathic surgery, Dentofacial deformities, Maxillary expansion, Ortodontia, Procedimentos cirúrgicos minimamente invasivos, Cirurgia Ortognática, Deformidades dentofaciais, Técnica de expansão palatina

## Abstract

**Objective:**

The purpose of this article is to present the MISMARPE technique, a new minimally invasive surgical procedure to treat maxillary transverse atresia in adult patients under local anesthesia and on an outpatient basis.

**Technique description:**

The technique consists of miniscrew-assisted rapid palatal expansion (MARPE) associated with a minimally invasive approach using maxillary osteotomies, latency and activation periods until the desired expansion is achieved. The present MISMARPE technique was performed in 25 consecutive cases with a success rate of 96%, yielding good skeletal outcomes with minimal trauma. The expander appliances, with their anchorage types, and a description of the surgical steps of the MISMARPE technique are presented.

**Conclusion:**

MISMARPE is a new and effective alternative for less invasive treatment of maxillary transverse deficiency in adults, compared to conventional surgery. Emphasis is placed on the importance of systematic and well-established protocols, for executing the procedures safely and predictably.

## RESUMO

**Objetivo:**

O objetivo deste artigo é apresentar a técnica MISMARPE, um novo procedimento cirúrgico minimamente invasivo para tratamento da atresia transversa da maxila em pacientes adultos, sob anestesia local e em regime ambulatorial.

**Descrição da técnica:**

A técnica consiste na expansão rápida da maxila assistida por mini-implantes (MARPE) associada a uma abordagem minimamente invasiva com osteotomias maxilares, períodos de latência e ativação até que a expansão desejada seja alcançada. A técnica MISMARPE foi realizada em 25 casos consecutivos, com uma taxa de sucesso de 96%, produzindo bons resultados esqueléticos, com trauma mínimo. Nesse artigo são apresentados os aparelhos expansores usados, com seus tipos de ancoragem e a descrição dos passos cirúrgicos da técnica MISMARPE.

**Conclusão:**

A MISMARPE é uma alternativa nova e eficaz para o tratamento menos invasivo da deficiência transversa da maxila em adultos, em comparação à cirurgia convencional. Ênfase é dada à importância de protocolos sistemáticos e bem estabelecidos para a execução dos procedimentos de forma segura e previsível.

## INTRODUCTION

Maxillary transverse deficiency is a common dentofacial deformity, often associated with dental crowding, narrowing of the palate, and crossbite. Maxillary atresia, with varying degrees of severity, can have significant functional repercussions.^1^ Conventional orthopedic expansion or rapid maxillary expansion (RME) is the treatment of choice for cases involving young patients with growth potential, i.e., those without complete maturation of the midpalatal suture. In such cases, the use of appliances like Haas or Hyrax can yield substantial transverse gains, correcting the existing transverse discrepancy.^2^

For skeletally mature patients with significant maxillary atresia, where dentoalveolar compensation is not recommended, the RME procedure is ineffective, due to the substantial resistance of the circummaxillary sutures to opening. The solution in these cases was extensive surgical procedures involving weakening the resistance pillars through osteotomies,^3^ ^,^ ^4^ a surgical technique known as surgically-assisted rapid palatal expansion (SARPE). This intervention must be performed in a hospital setting, under general anesthesia, and is considered a procedure with higher morbidity and costs.^5^ ^,^ ^6^ Another treatment option would be the transverse correction using orthognathic surgery, with segmental osteotomy (segmented Le Fort I osteotomy - a variation of the classic Le Fort I maxillary osteotomy),^7^ ^,^ ^8^ allowing a transverse correction of up to 7mm. The patients benefit by undergoing only one procedure under general anesthesia for the total correction of their deformity (transverse, anteroposterior, and vertical). However, in some patients, the presented transverse deformity does not allow for adequate treatment with maxillary segmentation alone, necessitating a more significant pre-maxillary expansion.

The Minimally Invasive SARPE was conceived to reduce surgical morbidity, and align with contemporary orthognathic surgery concepts. This technique was published by Hernández-Alfaro et al.^9^ in 2010, and consists of performing an outpatient surgical expansion under local anesthesia and intravenous sedation using a minimally invasive access in the maxilla. This corresponds to an incision of the mucosa extending from the lateral incisor to the lateral incisor (radically smaller surgical access than in the conventional SARPE).

Both SARPE techniques, conventional and minimally invasive, are performed with orthodontic devices, such as the Hyrax type, using dental anchorage. However, some dental complications, bone resorption, and relapses are reported with this type of anchorage, especially in more mature patients.^10^ ^,^ ^11^

The use of skeletal anchorage devices to assist in the treatment of maxillary atresia, known as miniscrew-assisted rapid palatal expansion (MARPE), was also first published in 2010.^12^ In this technique, transverse expansion occurs by means of skeletal anchorage, assisted by bicortical mini-implants in the palate, anchoring the expander appliance. Since then, the MARPE procedure has gained popularity and been extensively studied, demonstrating successful results when well indicated, with significant skeletal gains due to bone anchorage. However, the evidence found in the literature is still mainly related to young adults, no older than the fourth decade of life,^13^ ^-^ ^19^ with isolated reports of cases at an older age.^20^ ^,^ ^21^

The Minimally Invasive Surgical and Miniscrew-Assisted Rapid Palatal Expansion (MISMARPE) was first published in 2022,^22^ after several years of research.^23^ The technique was developed to target adults with maxillary atresia, combining the concepts of minimally invasive surgery with skeletal anchorage (MARPE), aiming to achieve maxillary expansion in adults with a reduction in surgical morbidity and patient costs.

Thus, the present article aims to present the protocol of the MISMARPE technique, with different types of appliances (bone-borne or tooth-bone-borne anchorage), describing the surgical technique and main results obtained.

## MISMARPE - ANCHORAGE AND APPLIANCE TYPES

**Figure 1:**
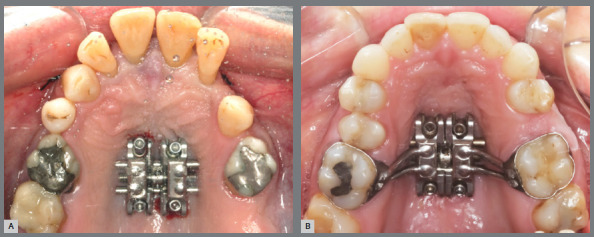
Images of bone-borne (A) and tooth-bone-borne (B) appliances used for performing the MISMARPE.

The MISMARPE technique is performed with expander appliances, using two types of anchorage: bone-borne (purely skeletal anchorage) and tooth-bone borne (also called hybrid anchorage, as it uses both dental and skeletal anchorage), as illustrated in Figure 1. The appliance consists of an expander screw (which can have different dimensions, according to the maxillary atresia) positioned on the palate with four bicortical miniscrews, two in the anterior region and two in the posterior region. The expander screw has a fixation bar that will be welded to the molar bands. The expander screw and the miniscrews are similar for both types of anchorage, maintaining the same minimally invasive osteotomy surgical protocol. In cases of exclusively skeletal anchorage, the fixation bar should be removed. The following sections will address the clinical and laboratory steps for fabrication, indications, advantages, and disadvantages of these two anchorage types, as well as a description of the surgical steps of the MISMARPE technique.

## REQUIRED EXAMS

A Cone Beam Computed Tomography (CBCT) is necessary for planning the appropriate location of the expander appliance and miniscrews. The Digital Imaging and Communications in Medicine (DICOM) images obtained are used for the 3D image reconstructions. Dolphin Imaging software (Dolphin Imaging & Management Solutions, Chatsworth, California, USA) was used to plan the cases described in this article. Intraoral scans of the upper arch and the patient’s palate should be acquired with an intraoral scanner and imported as STL files (a file format used to represent 3D models in computer graphics, originating from “stereolithography” or “standard tessellation language”). An STL file of the expander screw is also required.

**Figure 2:**
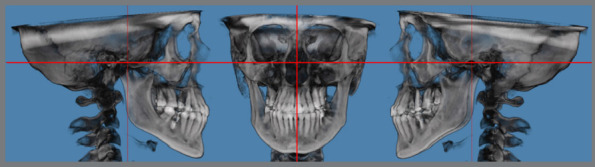
Orientation of the three-dimensional model from CBCT. The horizontal red line represents the Frankfurt plane, and the vertical red line represents the Nasio-Palatal line.

The first step is to orient the 3D tomographic image to standardize the head position from the Frankfurt plane (Or L-Po-Or R) parallel to the axial plane. The Nasion-Anterior Nasal Spine plane should be perpendicular to the Frankfurt plane (Fig. 2).

## TOOTH-BONE-BORNE ANCHORAGE

Tooth-bone borne anchorage, or hybrid expanders, feature skeletal anchorage using four parasutural mini-implants associated with bands cemented to the molars. The miniscrews are positioned to the body of the expander screw, while the wire extensions of the screw are welded to the bands of the first molars. The protocol for determining the positioning of the tooth-bone-borne expander screw can be performed with or without a guide. The following step-by-step process will detail the selection of the expander screw size and positioning, as well as the size of the miniscrews without the help of the positioning guide.

**Figure 3:**
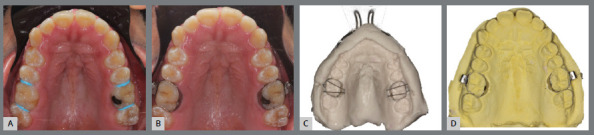
Separation (A) and bands selection (B) for the anchoring teeth of the tooth-bone-borne expander. (C) Transfer molding and obtained dental cast working model (D).

» Preparation of the anchoring teeth: Separate and band the upper first molars. Obtain an impression of the upper arch and palate, to produce a dental cast working model (Fig. 3).

**Figure 4:**
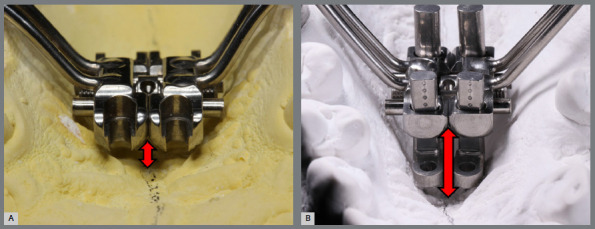
Positioning of the MARPE SL (**A**) and MARPE EX (**B**) expanders (PecLab, Belo Horizonte, Brazil). The adjustable extensions of the MARPE EX allow proper positioning of the expander and miniscrews in patients with extremely atresic palates.

» Selection of the expander screw size**:** It is recommended to use the largest possible expander screw size, respecting a distance of 1mm from the palatal mucosa and alveolar walls. The expander screw size can be chosen according to a template, presented in sizes of 6, 9, or 11mm (MARPE SL, PecLab, Belo Horizonte, Brazil). In cases of severe maxillary deficiencies and extremely narrow palates, adjustable expander screws (MARPE EX, PecLab, Belo Horizonte, Brazil) can be used. Adjustable extensions allow the miniscrews to be positioned close to the palate in patients with severe maxillary atresia. This screw is available in sizes 9, 11, or 13mm (Fig. 4).

**Figure 5:**
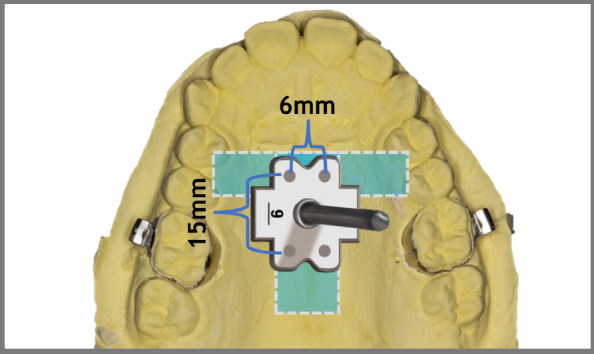
Template of the expander screw (PecLab, Belo Horizonte, Brazil) positioned in the “T” zone, recommended for installing the miniscrews.

» Definition of the expander screw position: The anteroposterior positioning of the expander screw on the palate must respect the most suitable area for miniscrew positioning, i.e., it should be allocated to a region of the palate with sufficient bone quantity. The “T” zone is interesting, due to the available alveolar bone and cortical thickness.^24^ ^,^ ^25^ This region is generally located in the parasutural region and the region of the third palatal rugae (Fig. 5). Therefore, the positioning of the anterior miniscrews should coincide with the third palatal rugae, equidistant from the midpalatal suture (3-mm distance on both the right and left sides). The posterior miniscrews should be positioned according to their respective template - about 15mm distal, according to the type of expander screw used (PecLab, Belo Horizonte, Brazil).

» Determination of the miniscrew size: The determination of miniscrew length should be individualized, to promote bicortical anchorage. Miniscrews that are too short can lead to expansion failure, due to their bending or destabilization. At the same time, too long miniscrews may penetrate into the nasal cavity, causing discomfort and otolaryngological problems. Therefore, miniscrew size should be defined with the aid of CBCT and digital planning software, such as Dolphin Imaging (Dolphin Imaging & Management Solutions, Chatsworth, California, USA) or CS 3D Imaging software (Carestream Dental, Atlanta, USA).

**Figure 6:**
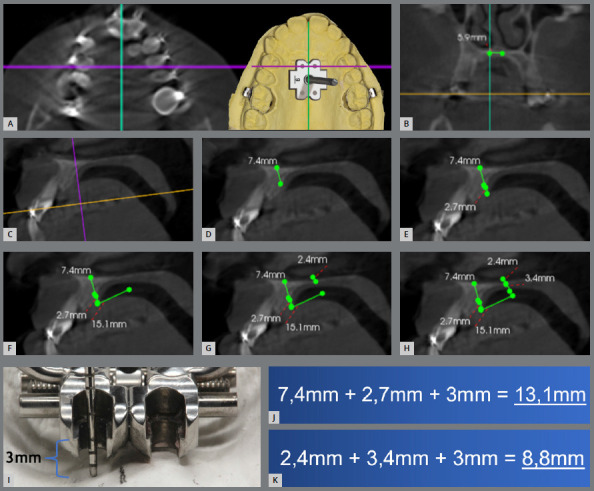
A) Definition of the anterior miniscrews position from the axial slice, considering the initially proposed marking on the working model. B) In the coronal slice, position the cursor 3mm away from the midpalatal suture, to select the miniscrews on the right and left sides. C) Sagittal view obtained to select the anterior miniscrews on the right side. D) Measurement of bone thickness, considering bicorticalization. E) Measurement of the palatal mucosa thickness. F) In this case, at a distance of 15mm distal, define the size of the posterior miniscrew. G) Measurement of the bone thickness of the posterior miniscrew on the right side, considering bicorticalization. H) Measurement of palatal mucosa thickness of the posterior miniscrew on the right side. I) To define the size of the miniscrew, add 1mm of palatal mucosa clearance + 2mm of expander thickness. J) Sum of distances for the selection of the right anterior and (K) right posterior miniscrews.

The first step involves transferring the anterior miniscrew positions, as defined in the working model, to the CBCT, in the axial slice. From this anteroposterior position, measure 3mm on each side of the midpalatal suture. At these points, in a sagittal slice, measure the bone thickness (from the nasal cortical to the palatal cortical) and the thickness of the palatal mucosa. Add 1mm to this measurement, referring to the distance from the expander appliance to the palate (if the expander has more than 1mm of separation from the palate, this distance should be considered in the total), and add the thickness of the expander body (2mm). It is suggested that the miniscrew size be chosen closest to the sum of these distances. The definition of the posterior miniscrew size follows the same protocol: measure the distance between the anterior and posterior holes, according to the template - in this case, 15mm distal. Figure 6 illustrates the steps of choosing the miniscrew size using the CBCT.

**Figure 7:**
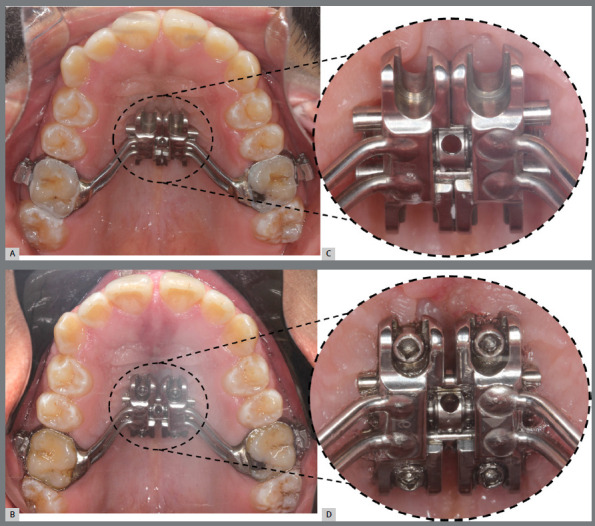
A) MARPE expander cemented. B) Installation of the miniscrews. In detail (C), observe the correct positioning of the expander in relation to the palatal mucosa, without pressure or ischemia in the region, and (D) the four miniscrews installed and well positioned in the expander holes.

» Installation of the expander appliance**:** The bands of the expander appliance should be cemented to the upper first molars (Fig. 7). Thus, in this protocol, the expander appliance serves as a guide for installing the miniscrews. The installation can be performed by the orthodontist, immediately after the cementation of the expander; or by the oral and maxillofacial surgeon, at the same time of the MISMARPE protocol.

## BONE-BORNE ANCHORAGE

**Figure 8:**
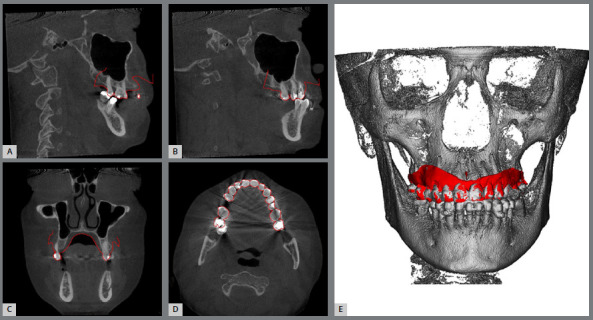
Superimposition of the maxillary STL model file on the CBCT images ( the red line is the external contour of the model ): A) right sagittal view; B) left sagittal view; C) coronal view; D) axial view; E) three-dimensional reconstruction, with the final position of the superimposed maxillary STL model.

**Figure 9:**
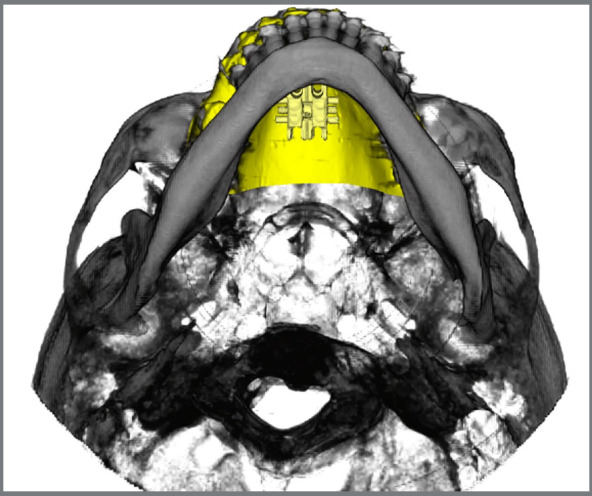
Screw positioned on the 3D reconstruction superimposed on the maxillary STL model.

**Figure 10:**
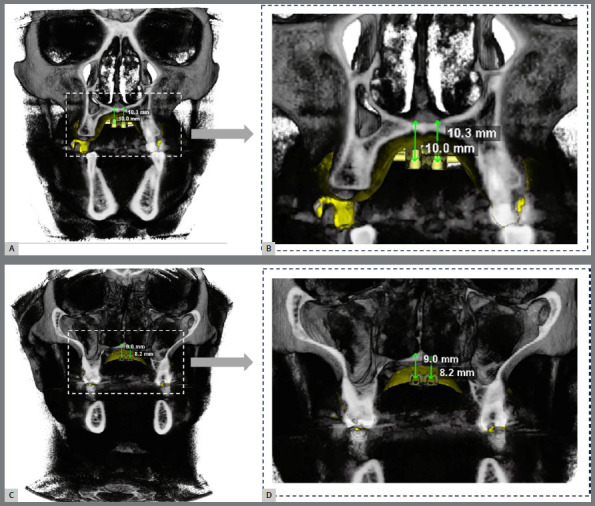
Measurements of the length of the miniscrews: **A** and B) anterior miniscrews; **C** and D) posterior miniscrews.

Bone-borne expanders feature skeletal anchorage using four parasutural miniscrews, without dental support. After the tomography’s spatial orientation, the upper arch’s STL file should be imported and superimposed onto the tomographic image. Use the sculpting tool to cut and facilitate positioning. The superimposition is based on the best fit of teeth in the axial, sagittal, and coronal planes (Fig. 8). The superimposition is saved, and the overlay tool is closed. After this step, the STL file of the expander screw is added and positioned at the desired location, respecting approximately 1.0 mm of distance from the contour of the palatal mucosa, using the file addition and positioning tool (Fig. 9). The miniscrews length, position, and insertion must be meticulously planned, to ensure ideal bicortical bone support and obtain the necessary primary stability to apply orthopedic forces. Precise virtual planning with CBCT, digital models, and surgical guides is fundamental^26^ to achieve these goals. The position of the expander screw is saved, and the file addition and positioning tool is closed. The length of the miniscrews is selected using the cutting tool. Select the three-dimensional model with the STL files and the image corresponding to the center of the screw slots. Firstly, move the cutting cursor to the region of the center of the anterior slots. Then, measure the distances between the slots’ bases and the nasal cavity’s internal cortical bone, to choose the appropriate length of the miniscrews (Fig 10). Afterward, repeat the same procedures for the posterior slots of the expander screw. To ensure that the miniscrews are bicortically anchored, 1 mm is added to the obtained value, as a safety margin (considering the distance from the appliance to the palatal mucosa).

**Figure 11:**
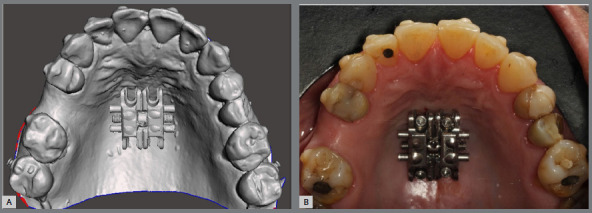
A) STL file of the upper arch with the screw virtually positioned B) Bone-borne appliance positioned.

» Positioning of the expander appliance: Using the Photo/Surfaces tool (Selection/Display), the digital maxillary model with the expander screw in the desired position is exported as a single STL file (Fig. 11A) and printed with a 3D printer. The model can be printed with an SLA (Stereolithography, Form 2; Formlabs, Somerville, Massachusetts, USA) or DLP (Digital Light Processing, SprintRay; Los Angeles, California, USA) printer. An acetate plate is laminated onto the printed model, to guide the virtual position of the expander screw on the patient’s palate. Currently, it is possible to print the surgical guide directly. The surgical guide with the expander is positioned, and the miniscrews (PECLAB, Belo Horizonte, Brazil) with the previously selected lengths are inserted manually or with the assistance of a surgical implant motor, anchoring the expander appliance (Fig. 11B). The expander appliance can be installed in advance by the orthodontist, or installed by the surgeon on the same day of the surgical stage of MISMARPE.

## RECOMMENDATIONS AND CARE

Inflammation of the palatal mucosa can destabilize the miniscrews and potentially cause procedure failure. Therefore, patients should be instructed regarding oral hygiene care. In addition to regular brushing, water jets should be performed using a 5 or 10ml syringe, directing the water jet between the expander and the palatal mucosa. Patients should also be advised to avoid hard and sticky foods (such as candies and chewing gum). The patient should apply 0.2% chlorhexidine digluconate gel twice a day, with the help of a brush.

After installation of the expander appliance, the surgical phase of the procedure is carried out.

## MISMARPE - SURGICAL PHASE

### PREOPERATIVE

The MISMARPE technique was designed to be performed in an outpatient setting, under local anesthesia, and without sedation assistance. However, for patient comfort, if necessary, an anxiety control protocol can be implemented, with the oral administration of a benzodiazepine for conscious sedation, 30 minutes before the surgical procedure, or intravenous sedation performed by an anesthesiologist.

### TECHNIQUE - STEP BY STEP

The oral cavity is antiseptically cleansed with 0.12% chlorhexidine, and sterile fields are set up for the surgical procedure. Local anesthetics are administered, using a potent anesthetic with a vasoconstrictor (typically, 4% articaine with 1:100,000 epinephrine): blocking of the posterior superior alveolar nerves, infraorbital nerves, and nasopalatine nerve, along with infiltration into the vestibular mucosa of the maxilla in the region of the upper labial frenulum, to aid in hemostasis and facilitate total mucoperiosteal detachment. The anesthetic technique is further complemented by infiltrations into the palatal mucosa in the paramedian region, where the four miniscrews of the MARPE expander will be installed.

**Figure 12:**
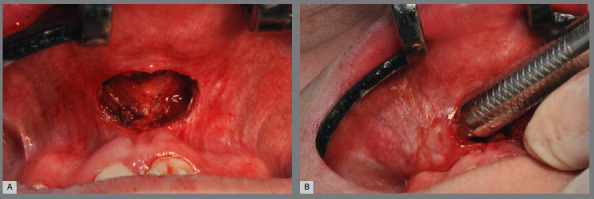
Minimally invasive approach to the maxilla: A) incision extending from tooth #11 to tooth #21; B) subperiosteal detachment through the tunnel.

The minimally invasive approach is performed through a mucoperiosteal incision in the anterior vestibular region of the maxilla, 3 mm above the mucogingival line. The surgical access averages 1 cm, extending from central incisor to central incisor (Fig. 12A). Subperiosteal detachment is then performed in the nasomaxillary region, without invading the nasal fossa and without detaching the musculature inserted into the anterior nasal spine, only accessing the entrance of the piriform aperture in its lower lateral portion on the left and right sides, and finally creating a tunnel-like detachment to the posterior region of the maxilla, bilaterally (Fig. 12B).

**Figure 13:**
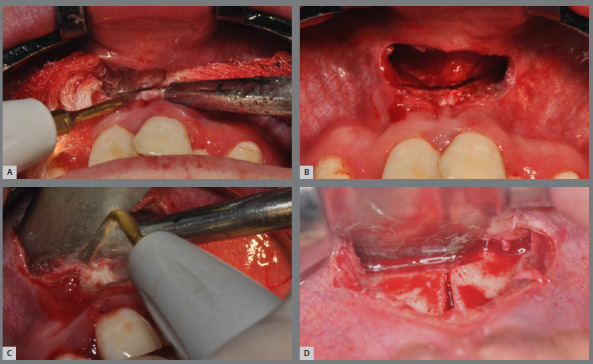
Subspinal osteotomy and vertical osteotomy in the midline: **A,** C) performing osteotomies with piezoelectric technology tip; **B**, D) clinical aspect after osteotomies.

The first osteotomy, called subspinal osteotomy, is performed at the anterior nasal spine. The anterior portion of the nasal mucosa is then detached from the nasal floor with a periosteal elevator (Fig. 13A, B). Afterward, a vertical osteotomy is performed in the midline between the central incisors, corresponding to the midpalatal suture, covering the vestibular and palatal bony cortical bones (Fig. 13C, D). Both osteotomies are carried out using piezoelectric technology.

**Figure 14:**
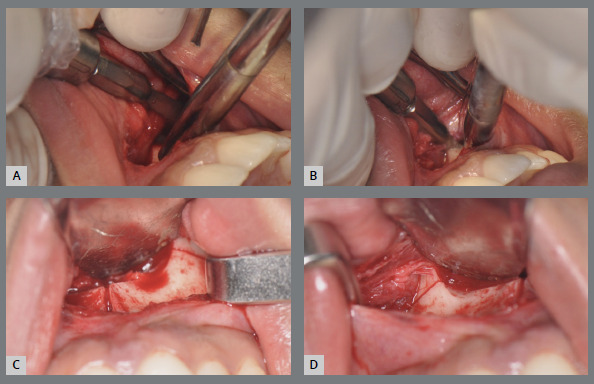
Horizontal maxillary osteotomy: **A, B**) execution with reciprocating cutting saw, on the left side; **C**) aspect of the maxilla after horizontal osteotomy (left side); **D**) aspect of the maxilla after horizontal osteotomy (right side).

Finally, a horizontal osteotomy of the maxilla is performed with a reciprocating cutting saw or piezoelectric tip. The region is visualized through the previously detached mucosal tunnel, with the help of the retraction of the vestibular mucosa. Thus, the cutting saw, or the piezo tip, is positioned in the posterior region at the level of the zygomatic pillar, just above the dental roots, and the medial walls of the maxillary sinuses are cut as the cutting saw progresses medially, up to the piriform aperture (Fig. 14A, B). The total extent of the cut is slightly oblique, with a more posterior lower inclination and a more anterior upper inclination, always respecting the limit above the dental roots. The same osteotomy is performed on the right and left sides, following the same criteria (Fig. 14C, D).

**Figure 15:**
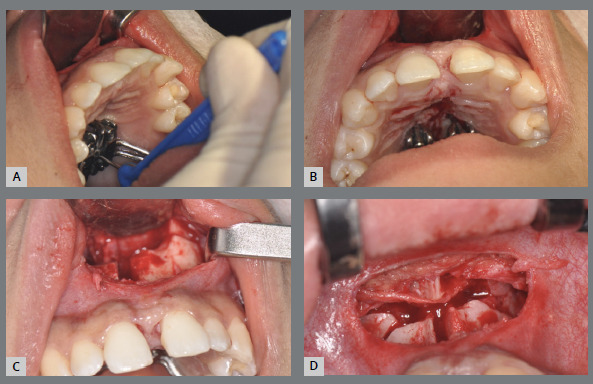
Expander screw activation in the intraoperative period: **A**) activation with the key; **B**) visualization of diastema between the upper central incisors; **C**) visualization of diastema and opening of osteotomy; **D**)aspect of vertical and horizontal osteotomies (bilaterally) after activation.

The MARPE expansion device is then activated until a small diastema between the central incisors is visualized. This step is essential to check the fragility of the bone resistance zones, especially in the midline (Fig. 15). Subsequently, the expander screw is closed again.

**Figure 16:**
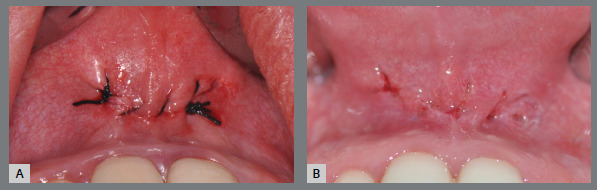
Final aspect of the operative wound: **A**) immediate postoperative - continuous suture with 4-0 silk thread; **B**) 7 days postoperative, after suture removal.

After reviewing hemostasis and abundant irrigation with saline solution, the operative wound is sutured. Single isolated stitches or continuous sutures can be performed using absorbable or non-absorbable suture thread (Fig. 16).

### POSTOPERATIVE CARE

Antibiotics, anti-inflammatory drugs, analgesics, and mouthwashes with a 0.12% chlorhexidine solution are prescribed for the postoperative period. Postoperative care also includes avoiding hot foods, refraining from suction movements, maintaining rest, avoiding exposure to heat and sunlight, and applying local ice in the initial days.

The MARPE device remains inactivated for 7 days, corresponding to a latency period. After this first week, the patient returns for clinical evaluation with the surgeon and suture removal. Subsequent follow-ups will continue with the orthodontist, for ongoing treatment.

### TECHNICAL CONSIDERATIONS

The MISMARPE surgical procedure, as described here, is the result of years of technical refinements by a surgeon and his experienced team in minimally invasive techniques. It is recommended that, for the execution of the MISMARPE, the oral and maxillofacial surgeon has the proper training to perform it safely. It is not a technique recommended for beginners, but for surgeons at a more advanced level, especially those experienced in minimally invasive orthognathic surgery. The smaller access to the maxilla provides a restricted view of the structures, so the professional must go through a learning curve and technical evolution, to perform the osteotomies correctly. The maxillary incision can initially be made slightly more extensive (from canine to canine or from lateral incisor to lateral incisor, for example), until the surgeon develops greater skills with the cutting saw or piezoelectric tip.

Osteotomy using the cutting saw proves to be faster and more precise in terms of execution; however, it exhibits greater vibration, causing more discomfort to patients when not under sedation. On the other hand, the piezo osteotomy, despite being slower, may be a safer option for execution, as there is a lower risk to soft tissues and less vibration from the equipment.

Intravenous sedation requires patient monitoring and should be performed by a multidisciplinary team. Despite adding an additional cost to the procedure, it can be an interesting alternative for some more anxious patients or those who prefer not to witness the procedure consciously.

### POSSIBLE COMPLICATIONS

Bleeding: As with any surgical procedure, there is a risk of bleeding. Although MISMARPE is a surgically-assisted maxillary expansion technique, the risk of bleeding is significantly reduced, due to the absence of pterygomaxillary disjunction.

Nasal bleeding: The nasal mucosa is a well-vascularized and sensitive region that may experience moderate bleeding during the operative procedure, typically manageable through compression techniques. The patient may have mild nasal bleeding (epistaxis) in the postoperative period of 7 days, and should be instructed to control it with gauze or use topical vasoconstrictor medication (nasal drops), along with other precautions to avoid bleeding episodes.

Dental root injuries: These may occur if horizontal and/or vertical midline maxillary osteotomies are not performed respecting the limits of the dental roots.

Periodontal injury: This can occur in the interincisal papilla region, especially if the vertical osteotomy in the midline is not performed correctly. It is recommended to perform a thin osteotomy with a piezo tip of less than 1mm thick, to reduce the chance of this type of complication.

Miniscrews failure or loss: This can result in the failure of maxillary transverse expansion and/or the need for the installation of new anchoring devices.

## MISMARPE - ACTIVATION AND RETENTION

The required amount of expansion should be planned before the surgery, aiming to identify the actual need for transverse expansion in each case. It is crucial to consider factors that may mask the transverse deficiency, such as compensations in the lower arch (excessive lingual inclination of lower teeth) or anteroposterior malocclusions (Class II or III) that can hinder the evaluation of the real transverse discrepancy.

The expansion screw activation should start 7 days after surgery, following a latency period. After this initial 7-day period, the activation protocol begins with a quarter turn in the morning and a quarter turn at night (equivalent to a quarter turn every 12 hours). This activation pattern continues until the detection of a midline diastema (typically occurring between the second and third day). Afterward, the activation continues with a quarter turn per day (a quarter turn every 24 hours) until the desired transverse correction is achieved.

Once the desired expansion is reached, activations are interrupted. For safety, the screw can be stabilized with a 0.30-mm ligature wire (0.012-in). The appliance should remain in position for at least 6 months, to allow complete bone reorganization in the midpalatal suture and osteotomy regions. After this phase, orthodontic treatment can proceed.

**Figure 17:**
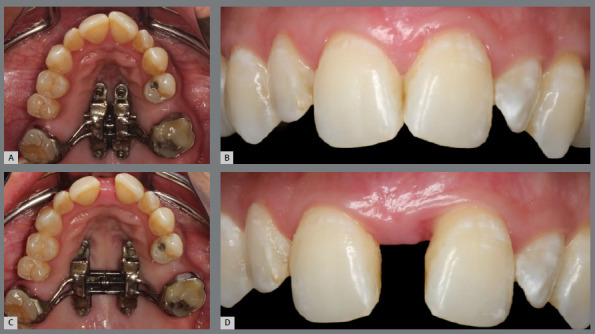
Adult patient with transverse maxillary deficiency, before (**A/B**) and immediately after (**C/D**) undergoing the MISMARPE protocol.

The diastema obtained immediately after expansion is broad (3 to 8mm), and may cause dissatisfaction to some patients (Fig 17). If necessary, resin can be added to the mesial surfaces of the teeth adjacent to the diastema (#11 and #21), ensuring a consistent separation, to enable the spontaneous return of the central incisors towards the midline during the expansion stabilization period.

## IMMEDIATE RESULTS ACHIEVED WITH THE MISMARPE TECHNIQUE

The MISMARPE technique was assessed in a clinical study (approved by the research ethics committee of the Pontifical Catholic University of Rio Grande do Sul, CAAE: 28064720.0.0000.5336), composed of 25 patients consecutively treated using the MISMARPE technique (12 with bone-borne expander and 13 with tooth-bone-borne expander).^23^ ^,^ ^27^ Of these, 24 patients successfully completed the procedure, with only 1 case of failure using the bone-borne expander, due to miniscrew stability issues. Therefore, the success rate of MISMARPE treatment was 96%.^27^ Data on the sample characteristics and patient distribution according to age group are presented in Table 1.

**Table 1:** Sample characteristics and patient distribution according to the age range of the 24 treated cases with MISMARPE (Source: modified from Piccoli^27^, 2023).

Age (years)	Skeletal anchorage (n)	Hybrid anchorage (n)	Total - n (%)
20-29	2	3	5 (20.8%)
30-39	3	7	10 (41.6%)
40-49	4	3	7 (29.1%)
50-56	2	0	2 (8.3%)
Total sample (n)	12	13	25
Median age			35.90 ± 9.52 years
Sex			10 males, 14 females

**Figure 18:**
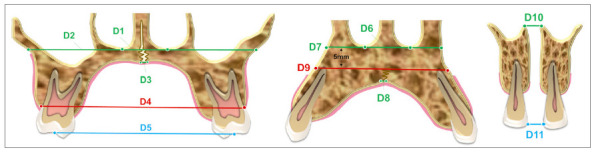
Illustration of the linear measurements evaluated pre and post-MISMARPE in the posterior region (first molars), anterior region (canines), and incisor region (Source: modified from Piccoli^27^, 2023).

After MISMARPE, with an average opening of the expansion screw of 7.78mm, there was a significant increase in all linear dental, alveolar, and skeletal transverse measurements.^27^ The anterior region of the maxilla showed more significant increases than the posterior region in all transverse and axial measurements assessed. Dental and alveolar measurements achieved greater transverse gains than skeletal measurements in the anterior and posterior regions.^27^ The result was a trapezoidal opening pattern, when observed in the coronal plane; and a “V” shape, when observed in the axial plane.^27^ Data regarding the average response after 7.78mm of activation are presented in Table 2 and Figure 18.

**Table 2:** Skeletal, alveolar, and dental measurements before and after MISMARPE (Source: Piccoli^27^, 2023).

Measure	Region	Initial (mm)	Post-MISMARPE (mm)	Difference (mm)
D1	Posterior nasal fossa floor width	17.14	19.68	2.54
D2	Maxilla posterior basal width	62.8	63.83	1.03
D3	Midpalatal suture - posterior region	0.34	3.6	3.26
D4	Posterior alveolar width of the maxilla	51.49	55.86	4.37
D5	Intermolar width	42.32	47.27	4.95
D6	Anterior nasal fossa floor width	10.15	14.21	4.06
D7	Maxilla anterior basal width	24.95	29.12	4.17
D8	Midpalatal suture - anterior region	2.63	8.18	5.55
D9	Anterior alveolar width of the maxilla	28.73	33.75	5.02
D10	Anterior nasal spine distance	0	6.05	6.05
D11	Interincisal distance	0.07	5.69	5.62

## DISCUSSION

Transverse maxillary deficiency in adults is a common malocclusion that requires correction. The evolution of dental materials and techniques has expanded the possibilities for orthodontic and surgical treatment of dentofacial disharmonies. Initially, treatment alternatives for transverse deficiency in adults were limited to camouflaging the transverse disharmony with dentoalveolar expansion of the upper arch, often accompanied by an increase in lingual inclination of the lower teeth, or by extensive surgical procedures. With the evolution of orthodontic and surgical techniques, new options for more effective and less invasive treatments have emerged and gained prominence.

In 2010, one of the first articles in orthodontic literature on the effects of MARPE treatment was published.^12^ The authors presented a clinical case of a 20-year-old adult patient with maxillary transverse deficiency and Class III with mandibular prognathism, treated by adding skeletal anchorage to the expander with palatal miniscrews. This created a robust structure sufficient to overcome the resistance of the median palatal and circummaxillary sutures. From then on, the maxillary expansion procedure without surgical assistance was expanded to adolescents and young adults. The benefits of MARPE include significant dentoalveolar and skeletal increases,^15^ ^,^ ^28^ ^,^ ^29^ with improved respiratory and sleep quality.^30^ Despite isolated clinical reports of MARPE success in mature adults,^20^ ^,^ ^21^ the ideal age range for its indication involves adolescents and young adults, as MARPE success rate is relatively high for individuals in this range (86.84% to 100%),^13^ ^,^ ^28^ ^,^ ^29^ ^,^ ^31^ ^-^ ^33^ but declines considerably for adults above 30 years (20%^33^ to 76%^32^).

On the other hand, MISMARPE has a high success rate in the treatment of mature adults (96%),^27^ even when performed on patients up to their sixth decade of life and with advanced stages of maturation of the median palatal suture (stages D and E).^27^ The excellent prognosis for expansion in adults and mature adults is due to the weakening of maxillary resistance pillars, by means of the four minimally invasive osteotomies, releasing resistance zones in zygomatic pillars and the piriform aperture pillars, but without releasing the pterygomaxillary pillar. Skeletal anchorage provided by the miniscrews assists in the maxillary expansion process and contributes to the optimal prognosis of the MISMARPE technique. This surgical approach is performed in an outpatient setting, under local anesthesia, in about 24 minutes, measured from the first incision to the closure of the operative wound.^22^ It is important to emphasize the need for appropriate training and preparation of the surgeon, who should be familiar with minimally invasive surgical techniques and have previous experience with orthognathic surgery - in other words, it is not a technique recommended for beginners without specific training.

An advantage of the MISMARPE technique over MARPE is its predictability in treating maxillary transverse deficiency in mature patients; and, as a disadvantage, the need for osteotomy assistance, along with increased costs and factors inherent to surgical procedures. To date, there are no reliable guidelines to predict the success of skeletal expansion in mature patients.^20^ Thus, the clinical attempt of orthopedic expansion is the only reliable method to confirm the possibility of sutural opening.^20^

With the MISMARPE technique, patients perceive the surgical procedure as less invasive, probably due to the absence of hospitalization and general anesthesia, coupled with shorter operating times and reduced incisions.^23^ All these factors contribute to patients’ better acceptance and tolerance of the MISMARPE procedure.

The efficacy and stability of SARPE and its indications are widely reported in the literature. Adults with maxillary atresia are candidates for SARPE, and now, also for MISMARPE.^34^ The main benefits of MISMARPE over SARPE include not requiring hospitalization or general anesthesia, presenting lower costs for patients, insurance plans, and the government public health policies.

Despite the excellent prognosis of MISMARPE in adults,^23^ ^,^ ^27^ the primary indication for this technique is individuals with maxillary atresia from the fourth decade of life onwards. Another indication is for patients who have unsuccessfully undergone the MARPE technique. These individuals can be immediately subjected to the MISMARPE procedure, without expander replacement, hospitalization, or general anesthesia.

## CONCLUSION

The development of new techniques, whether through adaptation or combination of existing procedures, to provide better results for patients is essential in Dentistry. This article aimed to present the MISMARPE technique, a new and effective alternative for less invasive treatment of maxillary transverse deficiency in adults (compared to conventional surgery) under local anesthesia and on an outpatient basis. Emphasis is placed on the importance of systematic and well-established protocols, for executing procedures safely and predictably.

## References

[1] McNamara JA (2000). Maxillary transverse deficiency. Am J Orthod Dentofacial Orthop.

[2] Cannavale R, Chiodini P, Perillo L, Piancino MG (2018). Rapid palatal expansion (RPE) meta-analysis of long-term effects. Orthod Craniofac Res.

[3] Bell WH, Epker BN (1976). Surgical-orthodontic expansion of the maxilla. Am J Orthod.

[4] Lines PA (1975). Adult rapid maxillary expansion with corticotomy. Am J Orthod.

[5] Suri L, Taneja P (2008). Surgically assisted rapid palatal expansion a literature review. Am J Orthod Dentofacial Orthop.

[6] Gogna N, Johal AS, Sharma PK (2020). The stability of surgically assisted rapid maxillary expansion (SARME) a systematic review. Craniomaxillofac Surg.

[7] Posnick JC, Adachie A, Choi E (2016). Segmental maxillary osteotomies in conjunction with bimaxillary orthognathic surgery indications - safety - outcome. J Oral Maxillofac Surg.

[8] Haas OL, Guijarro-Martínez R, Gil APS, Meirelles LS, Oliveira RB, Hernández-Alfaro F (2017). Stability and surgical complications in segmental Le Fort I osteotomy a systematic review. Int J Oral Maxillofac Surg.

[9] Hernandez-Alfaro F, Mareque Bueno J, Diaz A, Pagés CM (2010). Minimally invasive surgically assisted rapid palatal expansion with limited approach under sedation a report of 283 consecutive cases. J Oral Maxillofac Surg.

[10] Muñoz-Pereira ME, Haas-Junior OL, Meirelles LS, Machado-Fernández A, Guijarro-Martínez R, Hernández-Alfaro F (2021). Stability and surgical complications of tooth-borne and bone-borne appliances in surgical assisted rapid maxillary expansion a systematic review. Br J Oral Maxillofac Surg.

[11] Smeets M, Costa O, Eman S, Politis C (2020). A retrospective analysis of the complication rate after SARPE in 111 cases, and its relationship to patient age at surgery. J Craniomaxillofac Surg.

[12] Lee KJ, Park YC, Park JY, Hwang WS (2010). Miniscrew-assisted nonsurgical palatal expansion before orthognathic surgery for a patient with severe mandibular prognathism. Am J Orthod Dentofacial Orthop.

[13] Jia H, Zhuang L, Zhang N, Bian Y, Li S (2021). Comparison of skeletal maxillary transverse deficiency treated by microimplant-assisted rapid palatal expansion and tooth-borne expansion during the post-pubertal growth spurt stage. Angle Orthod.

[14] Oliveira CB, Ayub P, Ledra IM, Murata WH, Suzuki SS, Ravelli DB (2021). Microimplant assisted rapid palatal expansion vs surgically assisted rapid palatal expansion for maxillary transverse discrepancy treatment. Am J Orthod Dentofacial Orthop.

[15] Brunetto DP, Sant'Anna EF, Machado AW, Moon W (2017). Non-surgical treatment of transverse deficiency in adults using microimplant-assisted Rapid Palatal Expansion (MARPE). Dental Press J Orthod.

[16] Zong C, Tang B, Hua F, He H, Ngan P (2019). Skeletal and dentoalveolar changes in the transverse dimension using microimplant-assisted rapid palatal expansion (MARPE) appliances. Semin Orthod.

[17] Cantarella D, Dominguez-Mompell R, Mallya SM, Moschik C, Pan HC, Miller J (2017). Changes in the midpalatal and pterygopalatine sutures induced by micro-implant-supported skeletal expander, analyzed with a novel 3D method based on CBCT imaging. Prog Orthod.

[18] Ventura V, Botelho J, Machado V, Mascarenhas P, Pereira FD, Mendes JJ (2022). Miniscrew-Assisted Rapid Palatal Expansion (MARPE) an umbrella review. J Clin Med.

[19] Kapetanovic A, Theodorou CI, Bergé SJ, Schols JGJH, Xi T (2021). Efficacy of Miniscrew-Assisted Rapid Palatal Expansion (MARPE) in late adolescents and adults a systematic review and meta-analysis. Eur J Orthod.

[20] Kim H, Park SH, Park JH, Lee KJ (2021). Nonsurgical maxillary expansion in a 60-year-old patient with gingival recession and crowding. Korean J Orthod.

[21] Ceschi M, Riatti R, Di Leonardo B, Contardo L (2022). Skeletal expansion using a miniscrew-assisted rapid palatal expansion in a 50-year-old patient. Am J Orthod Dentofacial Orthop.

[22] Haas OL, Matje PRB, Rosa BM, Rojo-Sanchis C, Guijarro-Martínez R, Valls-Ontañón A (2022). Minimally invasive surgical and miniscrew-assisted rapid palatal expansion (MISMARPE) in adult patients. J Craniomaxillofac Surg.

[23] Matje PRB (2020). Expansão rápida da maxila assistida por mini implantes e osteotomia maxilar minimamente invasiva.

[24] Wilmes B, Ludwig B, Vasudavan S, Nienkemper M, Drescher D (2016). The T-Zone median vs. paramedian insertion of palatal mini-implants. J Clin Orthod.

[25] Baumgaertel S (2009). Quantitative investigation of palatal bone depth and cortical bone thickness for mini-implant placement in adults. Am J Orthod Dentofacial Orthop.

[26] Maino BG, Paoletto E, Lombardo L, Siciiani G (2017). From planning to delivery of a bone-borne rapid maxillary expander in one visit. J Clin Orthod.

[27] Piccoli VD (2023). Efeitos imediatos da expansão rápida da maxila assistida por mini-implantes e cirurgia minimamente invasiva.

[28] Lim HM, Park YC, Lee KJ, Kim KH, Choi YJ (2017). Stability of dental, alveolar, and skeletal changes after miniscrew-assisted rapid palatal expansion. Korean J Orthod.

[29] Park JJ, Park YC, Lee KJ, Cha JY, Tahk JH, Choi YJ (2017). Skeletal and dentoalveolar changes after miniscrew-assisted rapid palatal expansion in young adults a cone-beam computed tomography study. Korean J Orthod.

[30] Brunetto DP, Moschik CE, Dominguez-Mompell R, Jaria E, Sant'Anna EF, Moon W (2022). Mini-implant assisted rapid palatal expansion (MARPE) effects on adult obstructive sleep apnea (OSA) and quality of life a multi-center prospective controlled trial. Prog Orthod.

[31] Choi SH, Shi KK, Cha JY, Park YC, Lee KJ (2016). Nonsurgical miniscrew-assisted rapid maxillary expansion results in acceptable stability in young adults. Angle Orthod.

[32] Jesus AS, Oliveira CB, Murata WH, Suzuki SS, Santos-Pinto AD (2021). Would midpalatal suture characteristics help to predict the success rate of miniscrew-assisted rapid palatal expansion. Am J Orthod Dentofacial Orthop.

[33] Oliveira CB, Ayub P, Angelieri F, Murata WH, Suzuki SS, Ravelli DB (2021). Evaluation of factors related to the success of miniscrew-assisted rapid palatal expansion. Angle Orthod.

[34] Silva AV, Rosa BM, Matje PRB, Rizzatto SMD, Oliveira RB, Haas OL (2024). Effects of SARPE and MISMARPE on correction of transverse maxillary deficiency a preliminary comparative evaluation. Orthod Craniofac Res.

